# Clinical Efficacy of Telemedicine Compared to Face-to-Face Clinic Visits for Smoking Cessation: Multicenter Open-Label Randomized Controlled Noninferiority Trial

**DOI:** 10.2196/13520

**Published:** 2019-04-26

**Authors:** Akihiro Nomura, Tomoyuki Tanigawa, Tomoyasu Muto, Takafumi Oga, Yasushi Fukushima, Arihiro Kiyosue, Masaki Miyazaki, Eisuke Hida, Kohta Satake

**Affiliations:** 1 CureApp Institute Karuizawa Japan; 2 Innovative Clinical Research Center Kanazawa University Kanazawa Japan; 3 Department of Cardiology Kanazawa University Graduate School of Medicine Kanazawa Japan; 4 Graduate School of Public Health St. Luke’s International University Tokyo Japan; 5 CureApp Inc Tokyo Japan; 6 Shinjuku Research Park Clinic Tokyo Japan; 7 Fukuwa Clinic Tokyo Japan; 8 Tokyo-eki Center Building Clinic Tokyo Japan; 9 Miyazaki Respiratory Care Clinic Tokyo Japan; 10 Department of Biostatistics and Data Science Osaka University Graduate School of Medicine Osaka Japan

**Keywords:** smoking cessation, nicotine dependence, telecare, telemedicine, mHealth, digital therapeutics, mobile phone

## Abstract

**Background:**

Tobacco is a major public health concern. A 12-week standard smoking cessation program is available in Japan; however, it requires face-to-face clinic visits, which has been one of the key obstacles to completing the program, leading to a low smoking cessation success rate. Telemedicine using internet-based video counseling instead of regular clinic visits could address this obstacle.

**Objective:**

This study aimed to evaluate the efficacy and feasibility of an internet-based remote smoking cessation support program compared with the standard face-to-face clinical visit program among patients with nicotine dependence.

**Methods:**

This study was a randomized, controlled, open-label, multicenter, noninferiority trial. We recruited nicotine-dependent adults from March to June 2018. Participants randomized to the telemedicine arm received internet-based video counseling, whereas control participants received standard face-to-face clinic visits at each time point in the smoking cessation program. Both arms received a CureApp Smoking Cessation smartphone app with a mobile exhaled carbon monoxide checker. The primary outcome was a continuous abstinence rate (CAR) from weeks 9 to 12. Full analysis set was used for data analysis.

**Results:**

We randomized 115 participants with nicotine dependence: 58 were allocated to the telemedicine (internet-based video counseling) arm and 57, to the control (standard face-to-face clinical visit) arm. We analyzed all 115 participants for the primary outcome. Both telemedicine and control groups had similar CARs from weeks 9 to 12 (81.0% vs 78.9%; absolute difference, 2.1%; 95% CI –12.8 to 17.0), and the lower limit of the difference between groups (–12.8%) was greater than the prespecified limit (–15%).

**Conclusions:**

The application of telemedicine using internet-based video counseling as a smoking cessation program had a similar CAR from weeks 9 to 12 as that of the standard face-to-face clinical visit program. The efficacy of the telemedicine-based smoking cessation program was not inferior to that of the standard visit–based smoking cessation program.

**Trial Registration:**

University Hospital Medical Information Network Clinical Trials Registry: UMIN000031620; https://upload.umin.ac.jp/cgi-open-bin/ctr_e/ctr_view.cgi?recptno=R000035975.

## Introduction

Tobacco is a major public health concern and the biggest preventable cause of a variety of disorders such as cerebro- and cardiovascular diseases, malignant tumors, and chronic obstructive pulmonary disease [1,2]. In Japan, the estimated number of smokers is more than 20 million, and smoking is responsible for approximately 130,000 deaths per year [3]. Thus, reducing the prevalence of smoking would help prevent deaths from life-threatening diseases [4].

To help quit smoking, Japan provides a smoking cessation program for patients with nicotine dependence. This 12-week program mainly consists of face-to-face clinic visits, involving counseling with a primary physician, checking exhaled carbon monoxide (CO) concentration, and prescribing smoking cessation medications [5]. Face-to-face clinic visits enable physicians to directly perform counseling, physical examinations, and various tests in person. However, despite these intensive efforts to have patients complete the program, more than half of the program participants could not complete the entire program [6]. The majority of patients with nicotine dependence in Japan are typically employed men who are extremely busy and unwilling to spend a large portion of their day visiting the clinic to receive the smoking cessation program [6]. Program dropout is normally considered equivalent to smoking cessation failure [7]. Therefore, promoting dedication and completion of the smoking cessation program could be crucial to making these people succeed at quitting smoking.

Recently, telemedicine, defined as remote delivery of health care via the internet, was considered one of the useful methods for providing medical care to patients [8,9]. Telemedicine minimizes patients’ burden of visiting a health institution and waiting for consultations with their physicians. Telemedicine could also be suitable for delivering a smoking cessation support program. Considering the preliminary report that 75% of participants could complete the smoking cessation program when conducted via telemedicine, the requirement of regular face-to-face visits at a clinic might be the reason for the low completion rate of the smoking cessation program [10]. Therefore, telemedicine that enables the smoking cessation program participants to receive their regular counseling via the internet could have the potential to improve the overall smoking cessation success rate among patients with nicotine dependence by providing them easier access to the program. However, it remains uncertain whether telemedicine using an internet-based video counseling system is effective for delivering the smoking cessation program compared to the standard face-to-face clinical visits.

In this study, we tested the clinical efficacy and feasibility of telemedicine using internet-based Web counseling compared to a standard face-to-face clinical visit in the smoking cessation program among patients with nicotine dependence.

## Methods

### Trial Design and Participants

This trial was a randomized, controlled, open-label, multicenter, noninferiority trial. Details of the trial protocol have been described elsewhere [11]. In brief, participants in both arms underwent the smoking cessation program used in Japan [5]. For the telemedicine arm, the entire smoking cessation program was conducted remotely via an internet-based video counseling system, except for the first registration visit [12]. For the control arm, participants followed the standard smoking cessation program conducted through face-to-face clinic visits. Participants in both arms used the CureApp Smoking Cessation (CASC) system [13]. The primary outcome was a biochemically validated continuous abstinence rate (CAR) from weeks 9-12.

We recruited individuals with nicotine dependence from March to June 2018. We conducted follow-up for 24 weeks. Only the participants who met all the inclusion criteria were included; those who met any of the exclusion criteria were excluded [11]. Briefly, we included participants who were diagnosed with nicotine dependence (Tobacco Dependence Screener score ≥5 points) [14], had a Brinkman index ≥200, had the will to quit smoking immediately, agreed to undergo the smoking cessation program, and could use a smartphone. We excluded participants who had severe mental illness, could not tolerate the follow-up for 6 months, had used smoking cessation supplements or medication before the registration, planned to use any smoking cessation aids or to participate in any kind of smoking, or had regular clinic visits for diseases other than nicotine dependence planned within 12 weeks of registration.

Primary physicians at each clinic obtained written informed consent from all trial participants. We confirmed that clinics participating in this trial could provide the standard smoking cessation support program and had the necessary equipment to provide Web-based telemedicine (eg, WiFi access in the facility). We conducted this trial in compliance with the Declaration of Helsinki, Medical Device Good Clinical Practice guidelines, and all other applicable laws and guidelines in Japan. The trial protocol was approved by the Tokyo-Eki Center-Building Clinic institutional review board. We reported the trial according to CONSORT-EHEALTH (V 1.6.1). This trial was registered at the University Hospital Medical Information Network Clinical Trials Registry (UMIN000031620).

### Randomization

We used the stratified-block randomization (four blocks) method with a 1:1 allocation ratio to achieve equal assignment to two arms with stratification of the trial sites. Participants were allocated to either the telemedicine arm or the control arm. The randomization was performed by the staff at each participating clinic at the time of participants’ registration, using a computer-generated random sequence.

### Procedures

Participants allocated to the telemedicine arm received internet-based Web counseling for the smoking cessation program. Participants assigned to the control arm received the conventional face-to-face clinic visits for the smoking cessation program. Both arms also received the CASC smartphone app and a mobile exhaled CO checker during the trial period (24 weeks).

The standard smoking cessation program in Japan consists of five face-to-face clinic visits lasting for 12 weeks, including doctor consultations and exhaled CO checks at a registered institution or clinic [5]. All study participants visited their primary physicians at their first visit to confirm that they fully understood the trial protocol. At this visit, the physicians decided to prescribe appropriate smoking cessation medication, to provide guidance in accordance with the standard program procedure, and to provide participants with the CASC smartphone app integrated with a mobile CO checker.

Following the first visit, telemedicine participants were supposed to receive counseling via the internet-based video counseling system with a standardized telemedicine platform application [12] instead of visiting their clinics to see their primary physicians. As in the standard program, they met with their physicians via video counseling at each planned visit at weeks 2, 4, 8, 12, and 24. The control participants were supposed to visit their clinics at weeks 2, 4, 8, 12, and 24.

### Overview of CureApp Smoking Cessation

The CASC system was developed by CureApp, Inc. (Tokyo, Japan). Details of the system have been demonstrated elsewhere [13]. The CASC system consists of the CASC smartphone app [15], mobile exhaled CO checker, and Web-based personal computer for primary physicians. For the telemedicine arm, the primary physicians provided the app prescription code to the participants at their first visit in the outpatient clinics. The telemedicine participants downloaded the app through their smartphones; activated the app by entering the code; and keyed in their baseline data, motivation, and self-confidence regarding smoking cessation. The CASC smartphone app has four main components: (1) keeping a smoking cessation digital diary (filled in once a day), (2) lectures and educational videos helping its users to quit smoking, (3) interactive counseling by chat-bot, and (4) daily measurement and recording of exhaled CO concentration levels at home using the mobile CO checker. The Web-based personal computer software for the primary physicians provided a data-management app from patients’ CASC smartphone apps and advice for physicians to follow the national clinical guidelines.

### Outcomes

The primary outcome was the biochemically validated CAR from weeks 9-12, which was consistent with the previous clinical trial of varenicline in Japan [16]. This measure is defined as the percentage of individuals continuously not smoking (success) during the specified period. This study defined smoking cessation success as self-reported continuous abstinence as well as exhaled CO concentration ≤ 10 ppm during the given period [16]. For example, CAR from weeks 9-12 indicates that the patient achieved smoking cessation success if he/she self-reported continuous abstinence for 9-12 weeks and his/her exhaled CO concentration was ≤10 ppm at week 12. We also evaluated the following secondary outcomes: CAR from weeks 9-24; changes in the scores on the Mood and Physical Symptoms Scale (MPSS) [17] and the 12-item French version of the Tobacco Craving Questionnaire (FTCQ-12) [18]; the Kano Test for Social Nicotine Dependence (KTSND) score [19] at weeks 8, 12, and 24; Nicotine Dependence Cognition Scale (NDCS) score at weeks 12 and 24 [11]; and all adverse events during the trial.

### Sample Size and Inferiority Margin

From previous pilot studies of a CASC smartphone app, the difference in CAR from 9-12 weeks between the CASC smartphone app group (78%) and historical control groups (not using the app; 54%) is 24% [20-22]. Therefore, we hypothesized that the telemedicine group would not provide clinically worse CAR from 9-12 weeks compared to the control group (prespecified margin of 15% based on estimated 80% CARs in both groups) [11]. We calculated the required sample size as 114 (57 per each arm) based on the precision of estimate that the lower limit of the 95% CI of the difference between treatment effects exceeded the threshold amount of 15%. Therefore, we aimed to recruit at least 114 participants to allow for this sample size.

### Statistical Analysis

We compared all endpoints between the telemedicine and control groups. Baseline characteristics were described by means and SDs, medians and interquartile ranges (for continuous variables), or proportions (for categorical variables). We analyzed the primary outcome using the full analysis set (excluding participants who violated the inclusion or exclusion criteria). In case a patient discontinued an allocated treatment, the case was considered as smoking cessation failure. We compared CARs between telemedicine and the control groups using a logistic regression model with crude odds ratios (ORs). For all outcomes, summary statistics and group difference measures (eg, ORs by logistic regression or mean differences) were presented with 95% CIs. We also tested if the CAR from 9-12 weeks of telemedicine and the control condition varied by subgroups. We assessed interactions of CAR from 9-12 weeks with each variable (greater than vs less than the median for continuous variables) and calculated ORs with 95% CIs in each subgroup. We used the Wilcoxon signed-rank test for comparing the scores regarding nicotine dependence between baseline and at weeks 12 and 24 in each group. R version 3.4.1 (R Foundation for Statistical Computing, Vienna, Austria) was used for all the analyses.

## Results

**Figure 1:**
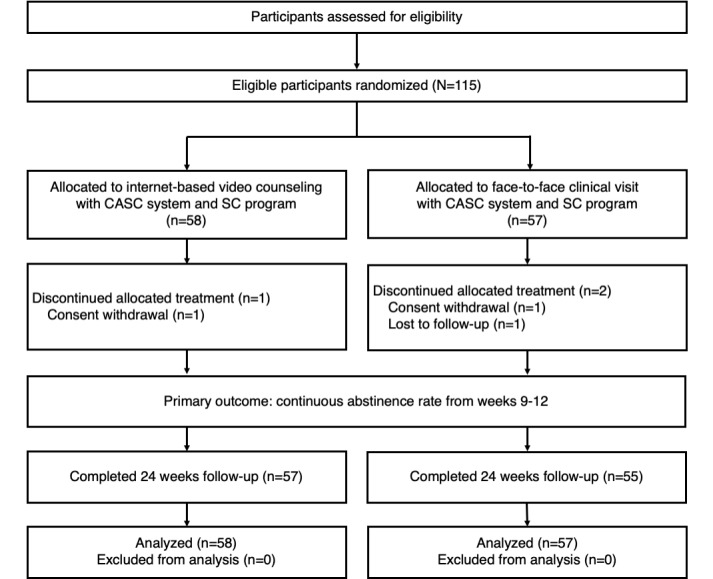
Trial flowchart. CASC: CureApp Smoking Cessation; SC: smoking cessation.

We randomized 115 participants to the telemedicine arm or control arm (Figure 1). Each arm was well balanced in the baseline characteristics (Table 1). All participants were prescribed smoking cessation medication at registration: varenicline for 55% and the nicotine patch for 45% of the participants. During the trial period, three participants discontinued their allocated treatment, two participants by consent withdrawal and one participant by loss to follow-up. Finally, all 115 participants were enrolled for further analyses.

Biochemically validated CARs from weeks 9-12 were 81.0% (95% CI 71-91) in the telemedicine group and 78.9% (95% CI 68-89) in the control group (Table 2). The absolute difference was 2.1% (95% CI –12.8 to 17.0); the lower limit of the 95% CI (–12.8%) was greater than the prespecified limit of –15% (Multimedia Appendix 1). The OR was 1.14 (95% CI 0.45-2.88). Moreover, CARs from weeks 9-24 were 74.1% (95% CI 63-85) in the telemedicine group and 71.9% (95% CI 60-84) in the control group. Therefore, there were no statistically significant differences between the telemedicine and control groups in CARs. In terms of program feasibility, adherence rates by session were both high (over 95%) during the trial (Multimedia Appendix 1). In addition, there were no serious or device-related adverse events in the groups during the trial (Multimedia Appendix 1). CASC product issues during the trial were reported by four participants (3.5%): two issues were related to mobile CO checker connection failure (one in telemedicine group and one in control group), one was related to login failure in the control group, and one was related to other malfunction of the primary physician interface (failure of a chart closure) in the telemedicine group.

Next, we demonstrated the efficacy of the telemedicine on CAR from weeks 9-12 by subgroup. Although the KTSND had a moderate interaction with the outcome, we found little evidence of significant interactions for any of the subgroup analyses (Multimedia Appendix 1).

We also assessed the evolution of scores by MPSS, FTCQ-12, KTSND, and NDCS (Multimedia Appendix 1). During the 24-week trial period, all FTCQ-12, KTSND, and NDCS scores were significantly decreased over time in both groups. MPSS scores, especially regarding urges (“time spent with urges” and “strength of urges”), were also significantly reduced over time in both groups.

**Table 1:** Baseline characteristics of the trial participants.

Characteristic		Total (N=115)	Telemedicine (N=58)	Standard care (N=57)
Age (years), mean (SD)		55 (11)	55 (12)	53 (10)
Male sex, n (%)		93 (81)	45 (78)	48 (84%)
Body mass index (kg/m^2^), median (interquartile range)		23 (21-26)	23 (21-26)	23 (21-25)
**Brinkman index, median (interquartile range)**		**480 (325-720)**	**500 (340-755)**	**450 (310-720)**
	Cigarettes per day	15 (13-20)	16 (15-20)	15 (12-20)
	Years of smoking	34 (27-40)	35 (29-41)	33 (26-38)
Number of attempts of smoking cessation before the trial, median (interquartile range)		1 (0-2)	1 (0-2)	0 (0-2)
TDS^a^ score, median (interquartile range)		7 (6-8)	7 (6-8)	7 (6-8)
FTND^b^ score, median (interquartile range)		5 (3-7)	5 (4-7)	5 (3-6)
KTSND^c^ score, median (interquartile range)		17 (16-20)	17 (16-20)	18 (15-21)
**Comorbidities, n (%)**				
	Hypertension	33 (29)	16 (28)	17 (30)
	Diabetes mellitus	9 (8)	5 (9)	4 (7)
	Dyslipidemia	42 (37)	23 (40)	19 (33)
**Medication, n (%)**				
	Varenicline	63 (55)	29 (50)	34 (60)
	Nicotine patch	52 (45)	29 (50)	23 (40)

^a^TDS: Tobacco Dependence Screener.

^b^FTND: Fagerström Test for Nicotine Dependence.

^c^KTSND: Kano Test for Social Nicotine Dependence.

**Table 2:** Continuous abstinence rates in percentages from weeks 9-12 (primary outcome) and weeks 9-24 (secondary outcome).

Continuous abstinence rate	Telemedicine, mean (SE)	Control, mean (SE)	Difference (95% CI)	Odds ratio (95% CI)
Weeks 9-12	81.0 (5.1)	78.9 (5.4)	2.1 (–12.8 to 17.0)	1.14 (0.45-2.88)
Weeks 9-24	74.1 (5.7)	71.9 (6.0)	2.2 (–14.0 to 18.4)	1.12 (0.49-2.57)

## Discussion

### Principal Results

In this randomized trial, we assessed the efficacy and feasibility of an internet-based remote smoking cessation program compared to the standard face-to-face clinical visit program among patients with nicotine dependence. We found that (1) CARs from weeks 9-12 were relatively high in both groups (81.0% in the telemedicine and 78.9% in the face-to-face control groups) and (2) the clinical efficacy of the telemedicine group was not significantly greater than that of the face-to-face control group in terms of CAR from weeks 9-12.

### Comparison with Prior Work

This trial has several important findings. First, CARs from weeks 9-12 were relatively high. CARs from weeks 9-24 also reached favorable results in both groups (74.1% in telemedicine and 71.9% in control). Compared to other countries [23-25], these CARs estimated using the Japanese smoking cessation program were higher. One of the main reasons could be that all participants in the Japanese smoking cessation program need to swear to quit smoking and sign a declaration of smoking cessation before participating in the program. This is a unique feature of the Japanese smoking cessation program, and it would strongly contribute to selecting highly motivated participants who want to quit smoking. In terms of previous reports from Japan, Nakamura et al reported CARs from weeks 9-12 and weeks 9-24 of 65.4% and 37.7%, respectively, in nicotine-dependent patients receiving 1 mg varenicline on prescription [16]. Japan’s Ministry of Health, Labour and Welfare also reported the latest success rate of 63.8% for smoking cessation at week 12 in nicotine-dependent patients undergoing the standard smoking cessation program [6]. Our trial showed comparable results with these studies, in terms of smoking cessation success rates. In addition, the efficacy of the telemedicine program was not inferior to that of the face-to-face clinic visit program over a prespecified limit value. In summary, telemedicine, or online-based video counseling, could be a viable alternative for managing a smoking cessation program, as it has shown a noninferior efficacy compared to the standard face-to-face clinic visit smoking cessation program.

Second, the dropout rates of the smoking cessation program were low even at week 24 in both groups, indicating potential improvement in the overall smoking cessation success rates. The Japanese national survey on the efficacy of nicotine-dependence treatment showed a linear relationship between the number of patient visits to outpatient clinics and the treatment success rate [6]. We used the CASC system for both groups in this trial. The system could cover intervals between counseling sessions or clinic visits that help patients obtain clinical guidance. It could also continuously monitor, promote, and encourage commitment to the smoking cessation program [13]. Thus, the CASC system, including the CASC smartphone app, might indirectly contribute to preventing dropouts from both groups.

Third, most of the scores for nicotine dependence in the trial were significantly decreased in both groups. This result indicated that the telemedicine-based and face-to-face smoking cessation programs with the CASC system were effective in improving the status of nicotine dependence and ameliorating craving for smoking over time in the smoking cessation program. However, mood symptoms (depressed, irritable in control, restless, hungry, and poor concentration) of the MPSS did not change during the trial. This outcome may be because the baseline median scores of these symptoms were already low (1=Not at all or 2=Slightly) and continuously stabilized during the trial period.

### Strengths and Limitations

The strength of this trial was that it was the first randomized controlled trial to test the efficacy and feasibility of telemedicine using internet-based video counseling directly compared with face-to-face clinical visit in patients with nicotine dependence. This trial had a few limitations. First, the prespecified limit value of 15% might not be conservative. However, CARs from weeks 9-12 in both groups were almost what we expected (both around 80%), and we considered 65% of CAR from weeks 9-12 to be a reasonable threshold for checking the clinical relevance of the telemedicine program compared with the face-to-face clinic visit program in accordance with previous reports [6,16]. Second, this Web- and smartphone-based program might not be applied for individuals who cannot buy or access mobile devices and did not have enough literacy to read or access the mobile technology. Third, concluding the efficacy of the telemedicine in this 3-months trial could be difficult. Further trials lasting longer than 3 months might be needed to confirm the long-term efficacy of telemedicine. Fourth, we did not collect data on adherence of smoking cessation medications that might affect the trial results.

### Conclusions

Telemedicine using internet-based video counseling for the smoking cessation program had a similar CAR from weeks 9-12 as that of the standard face-to-face clinic visit program. The efficacy of the telemedicine-based smoking cessation program was noninferior to that of the standard clinic visit–based smoking cessation program. The results of this trial demonstrated that internet-based counselling might be a viable alternative to standard clinic visits for smoking cessation.

## Appendix

Multimedia Appendix 1 Supplemental figures and tables.

Multimedia Appendix 2 CONSORT-EHEALTH checklist (V 1.6.1).

## Abbreviations

CAR: continuous abstinence rate
CO: carbon monoxide
FTCQ-12: 12-item French version of the Tobacco Craving Questionnaire
KTSND: Kano Test for Social Nicotine Dependence
MPSS: Mood and Physical Symptoms Scale
NDCS: Nicotine Dependence Cognition Scale
OR: odds ratio
