# Supplementary material for: Clinical Efficacy of Telemedicine Compared to Face-to-Face Clinic Visits for Smoking Cessation: Multicenter Open-Label Randomized Controlled Noninferiority Trial
Source: J Med Internet Res. 2019 Apr 26;21(4):e13520. doi: 10.2196/13520 (PMC6660118; doi:10.2196/13520)
Supplement: Multimedia Appendix 1 [file jmir_v21i4e13520_app1.pdf]

Clinical Efficacy of Telemedicine Compared with Face-to-face Clinic Visit on Smoking Cessation: A Multi-center, Open-label, Randomized Controlled, Non-inferiority Trial

Supplemental Files

Akihiro Nomura[1,2,3], Tomoyuki Tanigawa[1,4], Tomoyasu Muto[5], Takefumi Oga[6], Yasushi Fukushima[7], Arihiro Kiyosue[8], Masaki Miyazaki[9], Eisuke Hida[10], Kohta Satake[1,5]

[1] CureApp Institute, Karuizawa, Japan
[2] Innovative Clinical Research Center, Kanazawa University (iCREK), Kanazawa, Japan
[3] Department of Cardiology, Kanazawa University Graduate School of Medicine, Kanazawa, Japan
[4] Graduate School of Public Health, St. Luke's International University, Tokyo, Japan
[5] CureApp Inc., Tokyo, Japan
[6] Shinjuku Research Park Clinic, Tokyo, Japan
[7] Fukuwa Clinic, Tokyo, Japan
[8] Tokyo-eki Center Building Clinic, Tokyo, Japan
[9] Miyazaki RC Clinic, Tokyo, Japan
[10] Department of Biostatistics and Data Science, Osaka University Graduate School of Medicine, Osaka, Japan

Corresponding author:
Akihiro Nomura, MD, PhD
Innovative Clinical Research Center, Kanazawa University (iCREK)
Kanazawa, Japan
Phone number: +81-76-265-2049
E-mail: anomura@med.kanazawa-u.ac.jp

Supplemental figure 1. Absolute difference of CAR at weeks 9–12, 95% confidence interval and comparison to the pre-specified non-inferior margin.

The absolute difference of CAR at weeks 9–12 between the telemedicine and control groups was 2.1% (95% CI: -12.8–17.0). The lower limit of the 95% confidence interval of -12.8% was greater than the pre-specified limit of -15%.

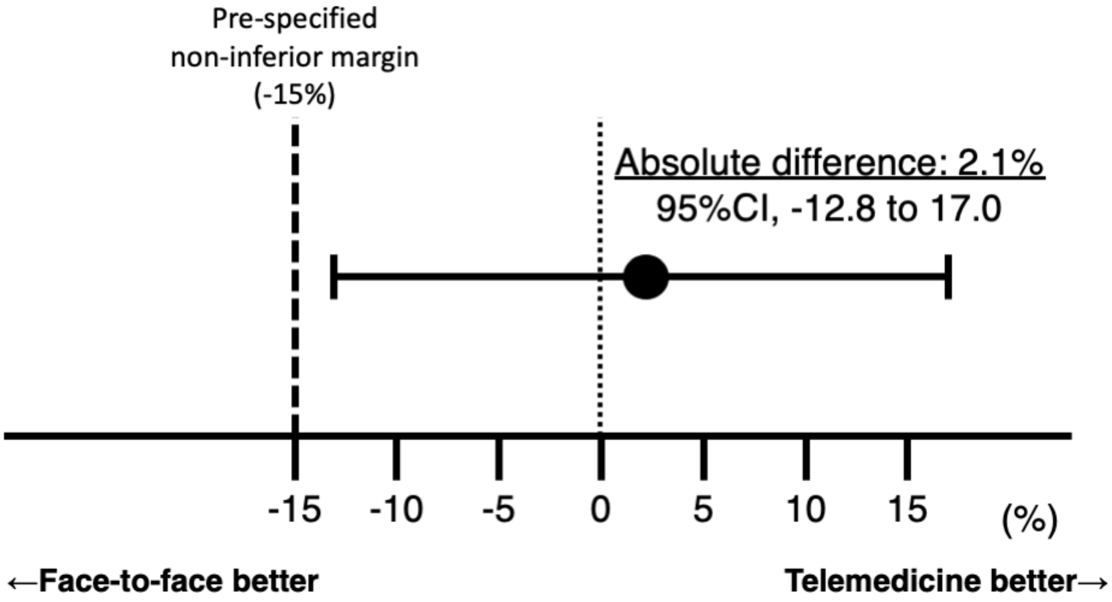

Supplemental figure 2. Efficacy of telemedicine on the continuous abstinence rates at weeks 9–12 by subgroup.

Subgroup		Telemedicine (se)	Control (se)	Odds ratio (95% CI)	P interaction
Age					0.44
	<50	72.0% (9.0)	76.7% (7.7)	0.78 (0.23-2.68)	
	≥50	87.9% (5.7)	81.5% (7.5)	1.64 (0.39-7.38)	
Sex					0.83
	Male	82.2% (5.7)	79.2% (5.9)	1.22 (0.43-3.51)	
	Female	76.9% (11.7)	77.8% (13.9)	0.95 (0.1-7.31)	
BMI					0.28
	<23	77.8% (8.0)	84.6% (7.1)	0.64 (0.14-2.55)	
	≥23	83.9% (6.6)	74.2% (7.9)	1.81 (0.53-6.73)	
Brinkman index					0.5
	<480	83.3% (7.6)	75.9% (7.9)	1.59 (0.42-6.84)	
	≥480	79.4% (6.9)	82.1% (7.2)	0.84 (0.22-2.98)	
Hypertension					0.43
	Yes	75.0% (10.8)	82.4% (9.2)	0.64 (0.11-3.48)	
	No	83.3% (5.8)	77.5% (6.6)	1.45 (0.48-4.5)	
Dyslipidemia					0.74
	Yes	82.6% (7.9)	84.2% (8.4)	0.89 (0.16-4.63)	
	No	80.0% (6.8)	76.3% (6.9)	1.24 (0.41-3.91)	
TDS					0.83
	<7	79.2% (8.3)	78.9% (9.4)	1.01 (0.22-4.49)	
	≥7	82.4% (6.5)	78.9% (6.6)	1.24 (0.38-4.21)	
FTND					0.5
	<5	82.6% (7.9)	75.0% (8.2)	1.58 (0.41-6.85)	
	≥5	80.0% (6.8)	82.8% (7.0)	0.83 (0.22-2.95)	
KTSND					0.03
	<17	68.2% (9.9)	87.0% (7.0)	0.32 (0.06-1.36)	
	≥17	88.9% (5.2)	73.5% (7.6)	2.88 (0.83-11.6)	
Medication					0.85
	Varenicline	89.7% (5.7)	88.2% (5.5)	1.16 (0.23-6.32)	
	Nicotine patch	72.4% (8.3)	65.2% (9.9)	1.4 (0.43-4.64)	

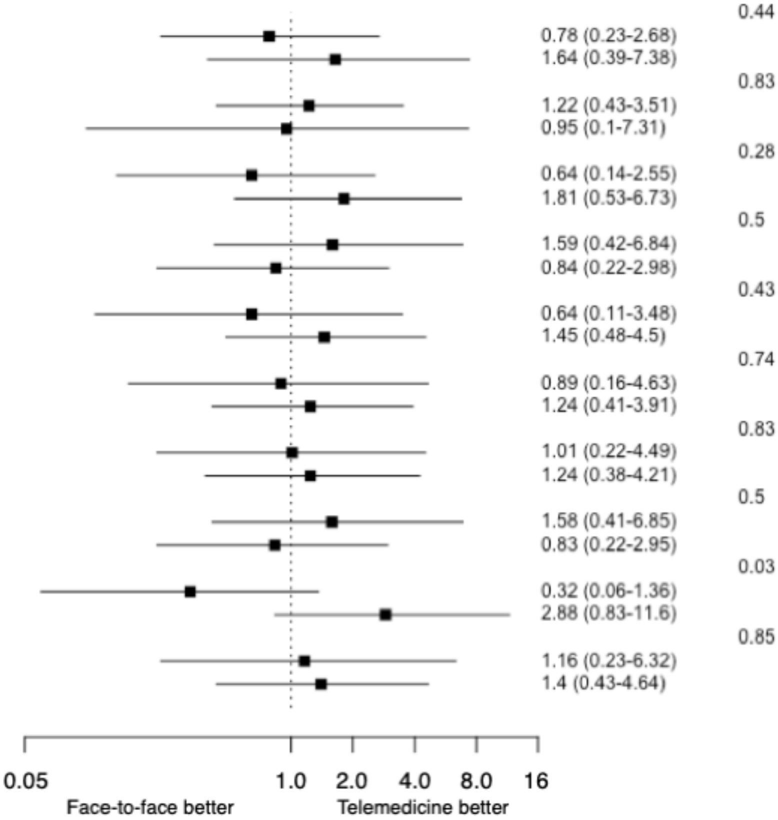

Abbreviations: BMI, body mass index; CI, confidence interval; FTND, Fagerström Test for Nicotine Dependence; KTSND, Kano Test for Social Nicotine Dependence; se, standard error; and TDS, Tobacco Dependence Screener.

Supplemental table 1. Program adherence rates by session.

	Total N	Weeks 2		Weeks 4		Weeks 8		Weeks 12		Weeks 24	
Standard care	57	55	96%	55	96%	55	96%	54	95%	55	96%
Telemedicine	58	57	98%	57	98%	57	98%	57	98%	57	98%

Supplemental table 2. List of adverse events.

	Total N = 115	Telemedicine n = 58	Standard care n = 57
At least one AE	25 (22)	12 (21)	13 (23)
Mild	3 (3)	2 (3)	1 (2)
Moderate	22 (19)	10 (17)	12 (21)
Severe	0	0	0
At least one AE related to trial device	0	0	0
At least one AE related to smoking cessation medication	22 (19)	10 (17)	12 (21)
At least one SAE	0	0	0

Values are n (%).
Abbreviations: AE, adverse event; and SAE, serious adverse event.

Supplemental table 3. Evolution of scores by MPSS, FTCQ-12, KTSND, and NDCS.

	Baseline (IQR)	2 weeks	4 weeks	8 weeks	12 weeks	24 weeks
MPSS						
Depressed						
Telemedicine	1 (1–2)	1 (1–2)	1 (1–2)	1 (1–2)	1 (1–2)	1 (1–1)
Control	1 (1–2)	1 (1–2)	1 (1–2)	1 (1–2)	1 (1–2)	1 (1–2)
Irritable						
Telemedicine	2 (1–2)	2 (1–3)	2 (1–3)	2 (1–2)	2 (1–2)	1* (1–2)
Control	2 (1–2)	2 (1–3)	2 (1–2)	2 (1–2)	1 (1–2)	1 (1–2)
Restless						
Telemedicine	1 (1–2)	2 (1–2)	1 (1–2)	1 (1–2)	1 (1–2)	1 (1–1)
Control	1 (1–2)	2 (1–2)	1 (1–2)	1 (1–2)	1 (1–1)	1 (1–2)
Hungry						
Telemedicine	3 (2–3)	2 (1–3)	2 (1–3)	2 (2–3)	3 (2–3)	2 (1–3)
Control	2 (2–3)	2 (1–3)	2 (1–3)	2 (2–3)	2 (1–3)	2 (1–3)
Poor concentration						
Telemedicine	2 (1–2)	2 (1–2)	2 (1–2)	2 (1–2)	1 (1–2)	1 (1–2)
Control	2 (1–2)	2 (1–2)	1 (1–2)	2 (1–2)	1 (1–2)	1 (1–2)
Time spent with urges						
Telemedicine	2 (2–3)	2 (2–3)	2 (1–2)	1 (1–2)	1* (1–2)	1* (0–2)
Control	2 (2–3)	2 (2–3)	2 (1–2)	2 (1–2)	1* (1–2)	1* (0–2)
Strength of urges						
Telemedicine	2 (1–3)	2 (1–2)	2 (0–2)	1 (0–1)	1* (0–1)	0* (0–1)
Control	2 (1–3)	2 (1–3)	2 (0–2)	1 (0–1)	1* (0–1)	1* (0–1)
FTCQ-12						
Emotionality						
Telemedicine	2.8 (2.1–3.8)	2.0 (1.5–2.8)	1.5 (1.0–2.4)	1.3 (1.0–2.0)	1.0* (1.0–1.8)	1.0* (1.0–1.8)
Control	3.0 (2.0–4.0)	2.5 (1.5–3.3)	1.8 (1.0–3.0)	1.3 (1.0–2.3)	1.0* (1.0–2.0)	1.3* (1.0–2.5)
Expectancy						
Telemedicine	5.0 (4.1–5.7)	3.7 (3.0–4.7)	3.3 (2.3–4.0)	2.7 (1.7–3.5)	2.0* (1.3–3.0)	2.3* 1.0–3.0)
Control	4.7 (4.0–6.0)	4.0 (3.0–4.7)	3.3 (2.3–4.3)	2.7 (1.7–3.7)	2.0* (1.3–3.7)	2.3* (1.7–3.3)
Compulsivity						
Telemedicine	3.3 (2.3–4.3)	2.0 (1.3–3.0)	2.0 (1.3–2.8)	1.7 (1.0–3.0)	1.7* (1.0–3.0)	1.3* (1.0–2.7)
Control	3.7 (2.3–4.0)	2.3 (1.7–3.0)	2.0 (1.3–3.0)	2.0 (1.0–3.0)	1.7* (1.0–2.7)	1.3* (1.0–3.0)
Purposefulness						

Telemedicine	5.5 (4.6–6.0)	4.5 (3.5–5.5)	4.0 (2.3–5.0)	2.5 (1.5–4.0)	2.0* (1.5–4.0)	1.5* (1.0–4.5)
Control	5.5 (4.5–6.0)	4.5 (4.0–5.5)	3.5 (1.5–5.5)	3.0 (1.5–4.5)	2.0* (1.0–3.5)	3.0* (1.0–4.3)
General Craving Score						
Telemedicine	3.8 (3.4–4.4)	2.9 (2.5–3.6)	2.6 (2.0–3.2)	2.1 (1.6–2.7)	2.0* (1.5–2.4)	1.8* (1.3–2.4)
Control	3.8 (3.2–4.6)	3.3 (2.7–3.6)	2.7 (1.8–3.3)	2.5 (1.6–3.1)	1.8* (1.4–2.6)	2.2* (1.4–2.8)
KTSND						
Telemedicine	17 (16–20)	-	-	12 (11–16)	11*(8–14)	11*(7–14)
Control	18 (15–21)	-	-	15 (12–18)	13*(9–17)	11*(7–16)
NDCS						
Telemedicine	12 (11–14)	-	-	-	6* (3–8)	6* (1–9)
Control	12 (10–14)	-	-	–	6* (4–9)	6* (3–8)

Abbreviations: FTCQ-12, 12-item French version of the Tobacco Craving Questionnaire; IQR, interquartile range; KTSND, Kano Test for Social Nicotine Dependence; MPSS, Mood and Physical Symptoms Scale; and NDCS, Nicotine Dependence Cognition Scale.
*P <0.001 by the Wilcoxon signed-rank test compared with the baseline in each group.
